# Preventive Dentistry

**Published:** 1922-02

**Authors:** A. S. Hopkins


### PREVENTIVE DENTISTRY

BY A. S. HOPKINS, D.D.S.

If prophylactic dentistry is ever to become a real vital force the dentist must educate his *clientèle.* Every time he looks into the patient's mouth he should not only see a filling, crown, or bridge, but should also see if the surrounding tissue shows the results of intelligent care.

From an artizan's standpoint, or the standpoint of the expert technician in the manner of replacing lost teeth, or restoring decayed ones, dentistry has made almost unbelievable strides, but from the standpoint of reducing and preventing dental decay and diseases of the peridental membrane, we have fallen down wofully.

Dare we compare our record of preventive medicine with that of the physician? Has he not practically eradicated smallpox, yellow fever, typhoid fever, etc.?

Some might be tempted to compare the service rendered by extracting teeth with so-called granulomas on the roots, as revealed by the X-ray, to the service of the physician, but is not this done usually to help cure some other disease? How much better it would have been to prevent that tooth from becoming liable to that granuloma.

We should also remember that that wonderful bridge or beautiful restoration depends upon the health of the tooth upon which it is placed; if it is not properly cared for the entire structure may be lost. Who would dare compare the bridge (regardless of its merits) with healthful functioning teeth? Who would dare compare that porcelain restoration, as beautiful as it is, with the perfectly formed, perfectly kept natural tooth? Is it not always, "That artificial tooth looks like a natural tooth"; never, "That natural tooth looks like an artificial one"? And yet how often we have seen the patient come in to have a tooth filled, and let him pass out with never a word of why.

Prophylactic dentistry may not be so wonderful from the technician's standpoint, but the results certainly should be more beneficial and gratifying than any other form of dentistry. Indeed, that dentist must feel a far greater degree of satisfaction who has brought the youth safely through the period of greatest susceptibility into manhood with all of his teeth sound and healthy, than the one who can show a nicely carved crown or a well-made bridge.

I do not mean to infer that we should not strive to better our technic of dentistry, but we should direct our efforts to the preventive.

The average patient can hardly afford the best restorative dentistry, but he *can* afford the *best* dentistry we can offer—*preventive dentistry.*

When this becomes a fact a new era will begin in the practice of dentistry and the dawn of the new day will beckon to a civilization unhampered with the scourge of decayed teeth.—Extract from *Cosmos,* January, 1922.

